# Inhalation of β2 agonists impairs the clearance of nontypable *Haemophilus influenzae *from the murine respiratory tract

**DOI:** 10.1186/1465-9921-7-57

**Published:** 2006-04-04

**Authors:** Nico A Maris, Sandrine Florquin, Cornelis van't Veer, Alex F de Vos, Wim Buurman, Henk M Jansen, Tom van der Poll

**Affiliations:** 1 Center of Infection and Immunity Amsterdam (CINIMA), Academic Medical Center, University of Amsterdam, Amsterdam, The Netherlands; 2 Center for Experimental and Molecular Medicine, Academic Medical Center, University of Amsterdam, Amsterdam, The Netherlands; 3 Department of Pathology, Academic Medical Center, University of Amsterdam, Amsterdam, The Netherlands; 4 Department of Pulmonology, Academic Medical Center, University of Amsterdam, Amsterdam, The Netherlands; 5 Department of Surgery, University of Maastricht, Maastricht, The Netherlands

## Abstract

**Background:**

Nontypable *Haemophilus influenzae *(NTHi) is a common bacterial pathogen causing human respiratory tract infections under permissive conditions such as chronic obstructive pulmonary disease. Inhalation of β2-receptor agonists is a widely used treatment in patients with chronic obstructive pulmonary disease. The aim of this study was to determine the effect of inhalation of β2 agonists on the host immune response to respiratory tract infection with NTHi.

**Methods:**

Mouse alveolar macrophages were stimulated in vitro with NTHi in the presence or absence of the β2 receptor agonists salmeterol or salbutamol. In addition, mice received salmeterol or salbutamol by inhalation and were intranasally infected with NTHi. End points were pulmonary inflammation and bacterial loads.

**Results:**

Both salmeterol and salbutamol inhibited NTHi induced tumor necrosis factor-α (TNFα) release by mouse alveolar macrophages in vitro by a β receptor dependent mechanism. In line, inhalation of either salmeterol or salbutamol was associated with a reduced early TNFα production in lungs of mice infected intranasally with NTHi, an effect that was reversed by concurrent treatment with the β blocker propranolol. The clearance of NTHi from the lungs was impaired in mice treated with salmeterol or salbutamol, an adverse effect that was prevented by propranolol and independent of the reduction in TNFα.

**Conclusion:**

These data suggest that inhalation of salmeterol or salbutamol may negatively influence an effective clearance of NTHi from the airways.

## Background

Chronic obstructive pulmonary disease (COPD) is frequently associated with exacerbations marked by increased dyspnea, wheezing, cough and increased sputum volume and purulence. Although the causes of such exacerbations are not always clear, bacterial infections of the lower airways may contribute substantially to morbidity, primarily as an evoking event or secondarily as a complication [1,2]. The bacteria most commonly isolated from patients are non typable *Haemophilus influenzae *(NTHi), *Streptococcus pneumoniae *and *Moraxella catarrhalis*, which account for 70 % of all exacerbations of COPD [1,3].

β2-receptor agonists are frequently used in the treatment of COPD. These agents induce bronchodilation via activation of β2-adrenoceptors on smooth muscle cells [4]. Apart from their presence on smooth muscle cells, β2-receptors are also found on cells involved in the regulation of inflammation like neutrophils, lymphocytes, monocytes and macrophages [5,6]. Stimulation of β2-receptors results in a number of anti-inflammatory effects, including inhibition of neutrophil activation and oxygen release, reduction of neutrophil-endothelial cell adhesion and a reduced capacity to release proinflammatory cytokines such as tumor necrosis factor (TNF)α and interleukin (IL)-1β by macrophages [5,6]. In line with these findings, we recently demonstrated that salmeterol, a long-acting β2-agonist, exerted anti-inflammatory effects in models of lipopolysaccharide (LPS)-induced lung inflammation in mice and humans, as reflected by a reduction in lung TNFα levels and an inhibition of neutrophil recruitment to the pulmonary compartment [7,8].

We hypothesized that β2 adrenergic agonists such as salmeterol or salbutamol would influence the clearance of NTHi from the respiratory tract. This hypothesis was based on two lines of evidence. First, the prompt influx of neutrophils into the lungs is important for the clearance of NTHi from the airways [9]. Thus, inhibition of neutrophil recruitment by salmeterol, such as observed during LPS-induced lung inflammation [7,8], may impair normal host defense. Second, indirect evidence indicates that TNFα plays a role in the protective immune response to NTHi, *i.e*. immunization with formalin killed NTHi resulted in more pronounced TNFα production which correlated with enhanced bacterial clearance [10]. Arguing that endogenous TNFα is of paramount importance for host defense against other bacterial respiratory pathogens. [11-14], we considered it conceivable that salmeterol-induced inhibition of TNFα production, such as found after pulmonary LPS challenge negatively impacts on the clearance of NTHi. Therefore, the present study was performed to determine the effects of salmeterol and salbutamol on the immune response to NTHi pneumonia.

## Methods

### Materials

Salmeterol and salbutamol were kind gifts from GlaxoSmithKline (Hertfordshire, United Kingdom). Propranolol (1 mg/ml) was obtained from Astra Zeneca (Zoetermeer, The Netherlands).

### Bacteriology

*H. influenzae *strain 12 (kindly donated by S.J. Barenkamp, St. Louis, MO) is a nontypable clinical isolate that has been used by our and other laboratories in investigations on murine pneumonia [15-18]. Classification, storage and inoculum preparation was performed as described before [17,18]. The inoculum contained 2 × 10^8 ^colony forming units (CFU) per ml. For each experiment, the number of CFU was determined by plating serial 10-fold dilutions on chocolate agar plates.

### Cell culture and stimulation

The murine alveolar macrophage cell line MH-S was obtained from American Type Culture Collection (ATCC CRL-2019; Rockville, MD). MH-S cells were cultured at 37°C in 5% CO_2 _in RPMI 1640 medium with 2 mM L-glutamine adjusted to contain 1.5 g/L sodium bicarbonate, 4.5 g/l glucose, 10 mM Hepes and 1.0 mM sodium pyruvate and supplemented with 10% FBS, 100 IU/ml penicillin, 100 μg/ml streptomycin and 0.05 mM 2-mercaptoethanol. For each experiment cells were seeded in 96-well plates (Greiner, Alphen a/d Rijn, The Netherlands) at a density of 0.5 × 10^5 ^per well and grown overnight. The next day cells were washed in medium and preincubated (5 minutes) with different concentrations of salmeterol or salbutamol (10^-5 ^– 10^-10 ^M) with or without propranolol (10^-5 ^M). Salmeterol and salbutamol were each dissolved to stock concentrations in PBS to which 2 droplets of glacial acetic acid were added. Dilutions of stock concentrations were made in MH-S medium. Control solutions were prepared similarly without the addition of salmeterol or salbutamol. Cells were stimulated with 5 × 10^5 ^heat killed (HK) NTHi (70°C for 30 minutes) and supernatants were collected after 3, 6, 12 and 24 h and stored at -20°C until measurement of TNFα.

### Mouse studies

Female C57BL/6 mice were purchased from Harlan Sprague Dawley (Horst, The Netherlands). At the start of the experiments mice were 8 weeks old. All experiments were approved by the Animal Care and Use Committee of the Academic Medical Center (Amsterdam, the Netherlands). Pneumonia was induced by intranasal inoculation of 50 μl (10^7 ^CFU) bacterial suspension as described before [17,18]; control mice received 50 μl sterile PBS. Mice were pretreated (at -30 minutes) with either control solution, salmeterol or salbutamol which were nebulized and inhaled. Salmeterol and salbutamol were each dissolved to stock concentrations in PBS to which 2 droplets of glacial acetic acid were added and dilutions were made in sterile 0.9% saline. Control solutions were prepared similarly without the addition of salmeterol or salbutamol. Inhalation of 1 ml control solution, salmeterol (2.4 mM) or salbutamol (2.4 mM) was achieved by attaching a plastic chamber (5 L) containing 8 conscious mice to an Aeroneb pro nebulizer (Medicare BV, Uitgeest, the Netherlands) as described [7]. Salmeterol and salbutamol treatments were repeated 6 or 12 hourly respectively until mice were sacrificed, while mice inhaled control solution in other groups or at time points they did not not receive β2-agonists. Propranolol (10 mg/kg) was injected i.p. 30 minutes before salmeterol treatment and repeated every 2 hours to block β-adrenoceptors. In a separate experiment mice were pretreated i.p. with 250 μg of a neutralizing anti-mouse TNFα monoclonal antibody (TN3) or mouse IgG1 (Chemicon, Temecula, CA) 3 hours before inoculation with NTHi; these mice inhaled salmeterol or control solution 30 minutes before NTHi infection as described above. TN3 is a well-characterized neutralizing anti-mouse TNFα monoclonal antibody that effectively neutralized endogenous TNFα in a variety of mouse models [19-22] including pneumonia [12,14].

### Determination of bacterial outgrowth

At 6, 12, 24 or 48 hours after infection mice were sacrificed after which lung and blood CFU were determined as described before [17,18].

### Bronchoalveolar lavage and flow cytometry

Bronchoalveolar lavage (BAL), total and differential cell count was performed as described [7]. The pellet was resuspended in FACS buffer (PBS supplemented with 0.5 % BSA, 0.01 % NaN_3_, and 0.35 mM EDTA) and expression of CD11b on neutrophils was determined by flow cytometric analysis as described previously, using rat anti-mouse CD11b PE and Gr-1 FITC (Ly-6G) antibodies (Pharmingen, San Diego, CA) [7].

### Histologic examination

In separate mice (n = 4 per treatment group at each time point) whole lungs were harvested for histologic examination 6 and 48 h after inoculation, fixed in 10 % formalin and embedded in paraffin. Sections of 4 μm were stained with hematoxylin and eosin, and analyzed by a pathologist who was blinded for the groups. Lung inflammation score was determined as described before [17,18].

### TNFα measurement and myeloperoxidase (MPO) assay

For TNFα measurement, lung homogenates were diluted 1:1 in lysis buffer (150 mM NaCl, 15 mM Tris, 1 mM MgCl.H_2_O, 1 mM CaCl_2_, 1 mM Triton X-100, 100 μg/ml Pepstatin A, Leupeptin and Aprotinin, pH 7.4) and incubated at 4°C for 30 minutes. Homogenates were centrifuged at 1500 × g for 20 minutes after which the supernatants were stored at -20°C until TNFα determination. TNFα was measured using a specific ELISA according to the manufacturers instructions (R&D Systems, Minneapolis, MN). The coefficient of variation was <10%. MPO activity in lung homogenates was measured as described previously [23].

### Statistical analysis

Values are expressed as mean ± SEM unless indicated otherwise. Differences between groups were analyzed with the nonparametric Kruskal-Wallis test followed by Mann Whitney *U *as posttest. P < 0.05 (two-sided) was considered statistically significant.

## Results

### Salmeterol and salbutamol inhibit TNFα production by mouse alveolar macrophages stimulated with heat killed NTHi

**Figure 1:**
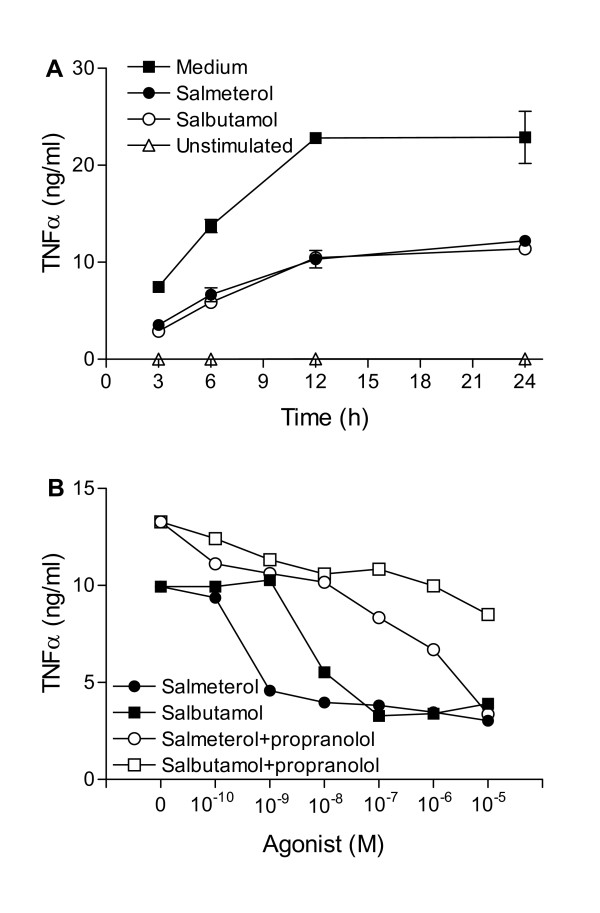
**Salmeterol and salbutamol inhibit TNFα production by mouse alveolar macrophages in vitro**. MH-S cells were incubated with 5 × 10^5 ^HKNTHi in the presence or absence of salmeterol or salbutamol. A, Effect of salmeterol and salbutamol (both 10^-7 ^M) on the kinetics of NTHi-induced TNFα release. B, Salmeterol and salbutamol inhibited NTHi-induced TNFα release (6 h incubation) in a dose dependent fashion, which can be reversed by propranolol (10^-5 ^M). Data are mean ± SEM of experiments performed in triplo. Please note that error bars fall within the symbols at multiple time points and concentrations. Markers of significance (described in the Results section) were omitted for reasons of clarity (figure 1B).

To determine whether β2 agonists inhibit TNFα release by alveolar macrophages stimulated with NTHi, MH-S cells were incubated with HKNTHi and supernatants were harvested after various time periods (figure 1A). Addition of 5 × 10^5 ^HKNTHi to mouse MH-S alveolar macrophages resulted in a rapid and sustained release of TNFα in culture medium reaching 13.73 ± 0.67 and 22.88 ± 2.67 ng/ml respectively after 6 and 24 h (figure 1A: p < 0.05 for NTHi stimulated vs unstimulated cells at all timepoints). NTHi-induced TNFα production was strongly inhibited by the β2-adrenoceptor agonists salmeterol and salbutamol (both 10^-7 ^M) at almost all incubation durations tested (figure 1A: p < 0.05 for salmeterol and salbutamol vs medium, except for salmeterol at t = 3 h). Inhibition of TNFα release by salmeterol or salbutamol occurred in a dose-dependent fashion (figure 1B). The inhibitory effect of both β2 agonists could be reversed by co-incubation with the β receptor blocker propranolol (10^-5 ^M) except for when very high doses of β2 agonists were used (figure 1B). Of note, stimulation of MH-S cells with NTHi in the presence of propranolol alone resulted in enhanced TNFα release (figure 1B).

### Salmeterol and salbutamol inhalation inhibit TNFα production in mouse lungs infected with NTHi

**Figure 2:**
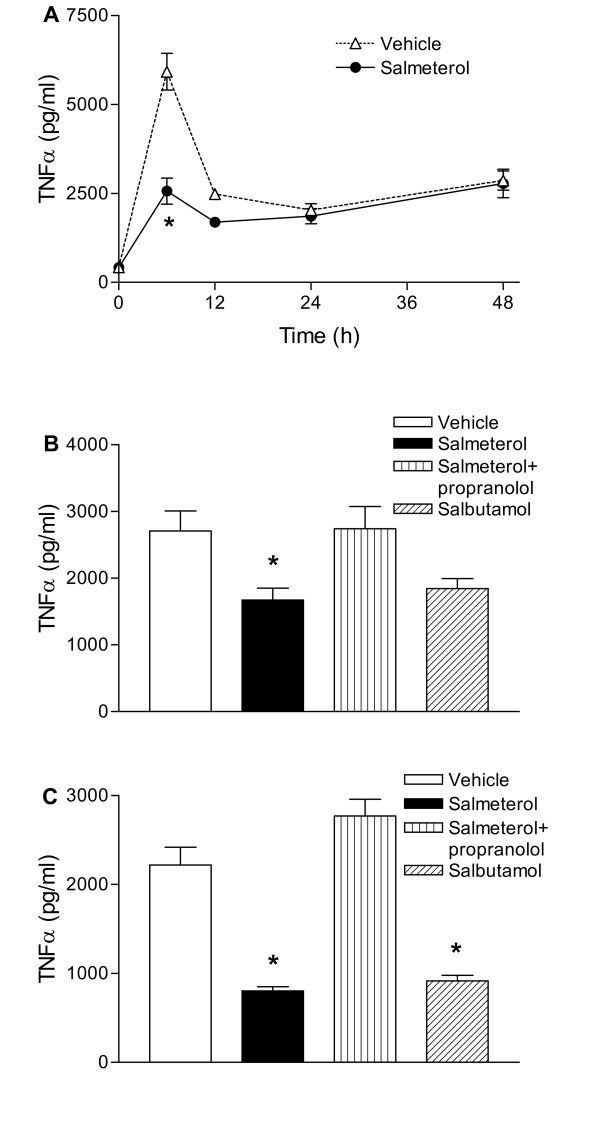
**Inhalation of salmeterol or salbutamol attenuate TNFα production in NTHi infected lungs in vivo**. Mice inhaled salmeterol or salbutamol before intranasal inoculation with 10^7 ^CFU NTHi. Some mice were injected with propranolol i.p. (10 mg/kg every 2 h). A, Salmeterol reduced TNFα production in NTHi infected lung homogenates 6 h post-challenge. The effect of salmeterol on TNFα production 6 h post-infection was mimicked by salbutamol and antagonized by propranolol in lung homogenates (B) and BALF (C). Values are mean ± SEM of 8 mice per group. * P < 0.05 versus vehicle and versus salmeterol + propranolol.

Intranasal inoculation of mice with 10^7 ^CFU NTHi significantly increased pulmonary TNFα concentrations, peaking after 6 hours (figure 2A: p < 0.05 versus non-infected mice at all time points). Inhalation of nebulized salmeterol reduced lung TNFα concentrations in NTHi infected mice, which reached significance at 6 hours post-challenge. The salmeterol induced reduction TNFα in lung homogenates and BALF at 6 hours post-infection was reversed by propranolol treatment (figure 2B and 2C: p < 0.05 versus salmeterol). Inhalation of salbutamol at equimolar concentrations as salmeterol also reduced TNFα in lung homogenates and BALF 6 h after infection with NTHi although in lung homogenates the difference with vehicle did not reach statistical significance (figure 2B and 2C: p < 0.05 versus vehicle in BALF).

### Salmeterol does not influence neutrophil influx or CD11b expression

**Figure 3:**
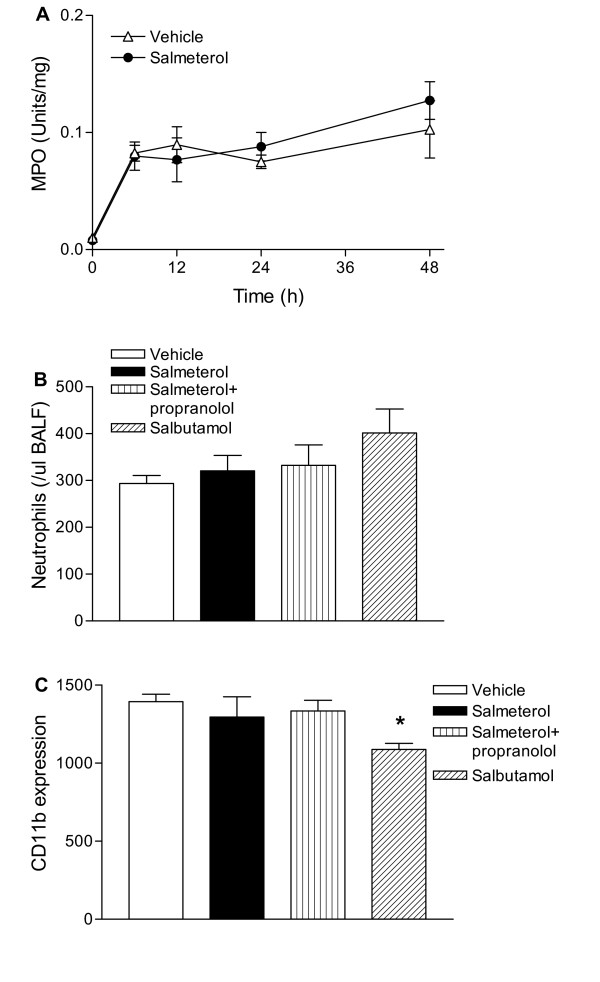
Inhalation of salmeterol or salbutamol does not modulate neutrophil influx. Mice inhaled salmeterol or salbutamol before intranasal inoculation with 10^7 ^CFU NTHi. Some mice were injected with propranolol i.p. (10 mg/kg every 2 h). A, Salmeterol did not alter neutrophil influx in NTHi infected lungs as determined by whole lung MPO activity. Additionally, no effect of salmeterol or salbutamol could be observed on neutrophil influx in BALF at 6 h after NTHi infection (B). C, Salbutamol but not salmeterol reduced CD11b expression on BALF neutrophils. Values are mean ± SEM of 8 mice per group. * P < 0.05 versus vehicle.

Intranasal inoculation of mice with 10^7 ^CFU NTHi resulted in a rapid pulmonary neutrophil influx (as measured by MPO activity) which was already strongly enhanced 6 hours post-infection, remaining high throughout the 48-hour observation period (figure 3A: p < 0.05 versus non-infected mice at all time points). Inhalation of nebulized salmeterol did not alter the neutrophil response to NTHi challenge. Additionally, neutrophil influx in BALF was strongly increased 6 hours after NTHi administration (p < 0.05 versus non-infected mice (data not shown)) which was not modulated by salmeterol or salbutamol. The expression of CD11b on neutrophils in BALF was not affected by salmeterol and was modestly but significantly decreased by salbutamol treatment (figure 3C: p < 0.05 versus vehicle).

### Salmeterol treated mice display unaltered pulmonary inflammation after NTHi challenge

**Figure 4:**
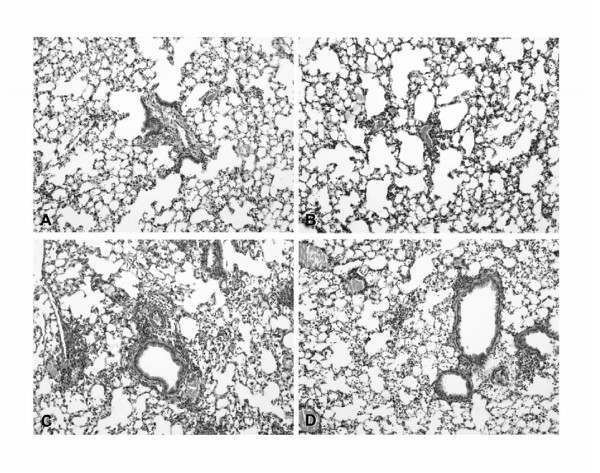
**Inhalation of salmeterol does not influence inflammation in NTHi infected lungs**. Mice inhaled vehicle (A, C) or salmeterol (B, D) before intranasal inoculation with 10^7 ^CFU NTHi. Mice (n = 4 per group) were sacrificed 6 (A, B) and 48 hours (C, D) post-infection and whole lungs were examined for inflammation. At 6 h post-infection, all mice displayed mild inflammation while at 48 h lung inflammation was more pronounced and diffuse. The inflammation scored did not reveal a difference between vehicle and salmeterol treated animals at both timepoints. H&E staining, magnification 10×.

Histologic examination of lungs at 6 hours post-infection revealed mild interstitial inflammation, edema and endothelialitis which were not different between vehicle and salmeterol treated mice (figure 4A and 4B). At 48 hours post-infection, lungs of mice displayed diffuse inflammation with moderate interstitial infiltrates, alveolitits, endothelialitis and pleuritis (figure 4C). The infiltrates consisted predominantly of granulocytes. Salmeterol treated mice showed unaltered pulmonary inflammation as assessed by the overall inflammation score which was 13.0 ± 2.1 and 10.5 ± 1.5 in vehicle and salmeterol treated mice respectively (Figure 4C and 4D). No difference in inflammatory cell type composition was observed after treatment with salmeterol.

### Salmeterol and salbutamol impair the clearance of NTHi from lungs

**Figure 5:**
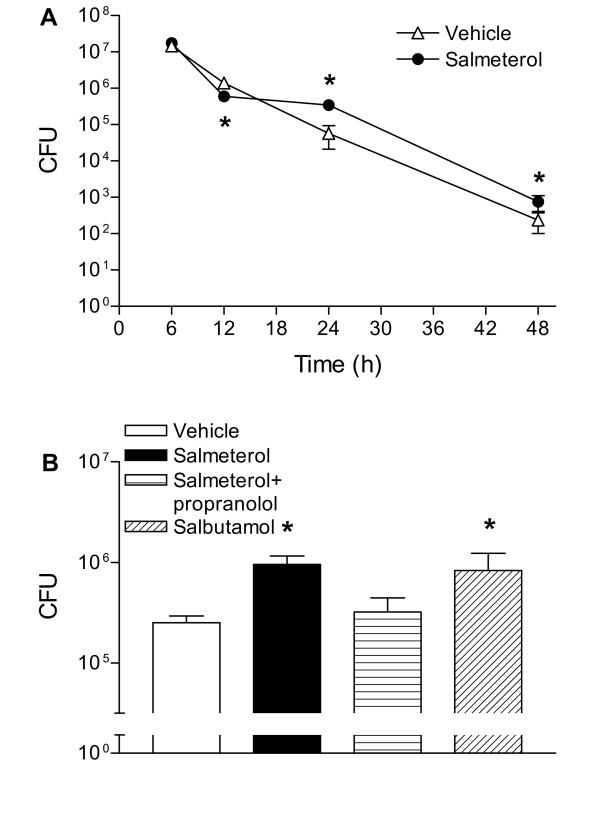
**Salmeterol and salbutamol impair the clearance of NTHi from lungs**. Mice inhaled salmeterol or salbutamol before intranasal inoculation with 10^7 ^CFU NTHi. Some mice were intraperitoneally injected with propranolol (10 mg/kg every 2 hours). A, Salmeterol inhibited bacterial clearance in infected lungs 24 and 48 h post-challenge. B, The effect of salmeterol on bacterial clearance 24 hours post-infection was mimicked by salbutamol and antagonized by propranolol. * p < 0.05 versus vehicle. Values are mean ± SEM of 8 mice per group.

To study the consequence of salmeterol inhalation for pulmonary anti-microbial defense the bacterial load was determined at various time points after NTHi infection. As reported earlier, intranasal inoculation of mice with 10^7 ^CFU NTHi did not result in lethality, with bacterial loads showing a gradual decline over several days [17,18]. Inhalation of salmeterol reduced the clearance of NTHi at 24 and 48 hours post-infection (figure 5A: p < 0.05 versus vehicle). At 6 hours no difference in bacterial clearance between control and salmeterol treated mice was observed, while at 12 hours post-NTHi bacterial load in salmeterol mice was slightly decreased (figure 5A). Although at this 12-hour time point difference was statistically significant, the biological significance is likely to be low, especially since we repeatedly found higher bacterial loads in mice treated with salmeterol at 24 hours post infection. Indeed, in a separate experiment, inhalation of salmeterol again was associated with more NTHi CFU in lung homogenates 24 h postinfection (figure 5B: p < 0.05 versus vehicle), an effect that was reversed by propranolol (figure 5B: p < 0.05 versus salmeterol). Moreover, also salbutamol inhalation resulted in an enhanced pulmonary bacterial load at this time point. In this model of pneumonia, blood cultures remained sterile at all time points.

### TNFα is not essential for the clearance of NTHi from mouse lungs

**Figure 6:**
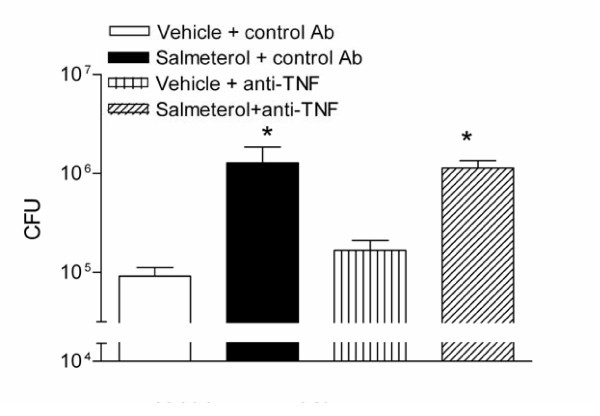
**TNFα is not essential to clearance of NTHi from the lungs**. Mice received an injection with an anti-TNF or control antibody (both i.p.), and inhaled salmeterol or vehicle before intranasal inoculation with 10^7 ^CFU NTHi. Salmeterol but not anti-TNF inhibited bacterial clearance in infected lungs 24 h post-challenge. * p < 0.05 versus vehicle. Values are mean ± SEM of 8 mice per group.

One obvious explanation for the decreased bacterial clearance observed in salmeterol treated mice was the inhibited production of TNFα. Therefore, mice received an anti-TNFα or matched control antibody prior to inhalation of salmeterol or vehicle (figure 6). In this experiment, salmeterol again increased the bacterial load at 24 hours post-infection (p < 0.05 versus vehicle). Remarkably, anti-TNFα did not influence the number of NTHi CFU in lungs of mice treated with either salmeterol or vehicle, indicating that TNFα was not essential for the bacterial clearance in NTHi infected lungs.

## Discussion

*H. influenzae *is a Gram-negative pathogen that frequently colonizes human respiratory mucosa. Nontypable strains are responsible for the majority of clinical disease caused by *H. influenzae *in the airways, and in particular patients with COPD, bronchiectasis and cystic fibrosis are susceptible to infection with NTHi [24]. We here tested the hypothesis that inhalation of β2 agonists, a treatment often given to patients with COPD and other chronic pulmonary disorders that predispose subjects to NTHi infection, would negatively influence host defense against this bacterium. Our results provide evidence that inhalation of either salmeterol or salbutamol indeed impairs the clearance of NTHi from the mouse respiratory tract in vivo.

Our hypothesis was, in part, based on our recent studies that investigated the effect of salmeterol on LPS-induced lung inflammation [7,8]. In these studies it was established that inhalation of salmeterol attenuated neutrophil influx into lungs after intrapulmonary delivery of LPS, concurrently reducing pulmonary TNFα concentrations. Here, we demonstrated that both salmeterol and salbutamol dose-dependently inhibit TNFα release by mouse alveolar macrophages stimulated with NTHi in vitro and that inhalation of either β2 agonist was associated with lower TNFα concentrations in lung tissue and BALF during NTHi pneumonia in vivo. The β2 agonist induced inhibition of TNFα release could be reversed by propranolol, indicating that the effect of these agents is mediated by β adrenergic receptors. Earlier studies reported on the systemic effects of β adrenergic agonists on TNFα release into the circulation after systemic (intravenous or intraperitoneal) administration of LPS [25-27].

We considered it conceivable that the reduced TNFα levels in β2 agonist treated mice was at least in part responsible for the impaired clearance of NTHi from the lungs. This assumption was based on various earlier findings. First, inhibition of TNFα in murine models of pneumonia caused by several respiratory pathogens, including *Klebsiella pneumoniae *and *Streptococcus pneumoniae*, resulted in a strongly enhanced bacterial outgrowth. [11-14]. Second, immunization with formalin killed NTHi accelerated bacterial clearance which was accompanied by increased TNFα production [10]. However, the present data clearly establish that TNFα does not contribute to an effective clearance of NTHi from the airways. The same anti-TNFα antibody that strongly impaired host defense during pneumonia caused by *S. pneumoniae *[12] or *K. pneumoniae *[14] did not influence the bacterial load during NTHi pneumonia. Moreover, anti-TNFα treatment did also not alter the effect of inhaled salmeterol on NTHi clearance. Interestingly, it was previously reported that mice deficient for the type I TNFα receptor displayed a modestly enhanced early clearance of *Pseudomonas aeruginosa *from the lungs during acute pneumonia [28]. Altogether these investigations suggest that early TNFα production in the lung is important for limiting the outgrowth of respiratory pathogens in the pulmonary compartment (i.e. *S. pneumoniae *and *K. pneumoniae *multiply in the mouse lung), whereas locally induced TNFα is of little importance for the immune response against bacteria that are cleared from the lungs (i.e. *P. aeruginosa *and NTHi) ([28] and the present study). A similar paradoxical role in murine pneumonia has been found for another prototypic proinflammatory cytokine IL-1, which facilitates host defense against *S. pneumoniae *[13,29], while having a modest negative impact on the clearance of *P. aeruginosa *[30]. It should be noted that in the current study we did not directly determine the capacity of anti-TNFα to neutralize TNFα activity in the lungs of mice infected with NTHi. Therefore, definitive proof that TNFα does not play a role in host defense against NTHi in this model is not provided. To obtain more insight into the role of TNF in host defense against NTHi pneumonia studies using TNFα or TNF receptor deficient mice are warranted. In addition, the potential protective effect of exogenous TNFα in mice treated with salmeterol could be evaluated using adenoviral TNFα gene transfer.

Neutrophils play an important role in the clearance of NTHi from the respiratory tract [9]. Neither salmeterol nor salbutamol influenced the recruitment of neutrophils to the lungs after infection with NTHi, as reflected by an unaltered number of neutrophils in BALF and in lung tissue slides, as well as by a similar rise in lung MPO concentrations in mice treated with salmeterol. This finding contrasts with the strong effect of salmeterol on influx of neutrophils into the pulmonary compartment after local delivery of LPS [7,8]. Notably, neutrophil emigration from the pulmonary circulation during inflammation caused by Gram-negative stimuli relies largely on expression of the β2 integrin CD11b/CD18 at the surface of neutrophils [31]. We recently demonstrated that salmeterol reduces CD11b expression on neutrophils recruited to the lung after intranasal administration of LPS and that blocking CD11b on neutrophils reproduces the inhibition of neutrophil influx by salmeterol treatment [7]. This led us to conclude that the effect of salmeterol on neutrophil influx during LPS-induced lung inflammation was at least in part due to a salmeterol-induced reduction in neutrophil CD11b expression [7]. In the present study, salmeterol did not influence neutrophil CD11b expression during NTHi infection, whereas salbutamol only had a modest effect. We do not have a firm explanation for these apparently different effects of salmeterol and salbutamol on neutrophil CD11b, although clearly the effect of salbutamol is weak and of doubtful biological relevance. Conceivably, this lack of a strong effect on neutrophil CD11b at least in part explains the present finding that inhalation of β2 agonists did not affect neutrophil trafficking to the lung. Further studies are warranted to assess whether the impact of β2 agonists on neutrophil functions only applies to sterile stimuli eliciting a brisk but transient inflammatory response in the lung (e.g. such as induced by LPS).

Salmeterol achieves instantaneous topical concentrations at least as high as 1 μM in human lung [32]. In vitro, salmeterol and salbutamol inhibited TNFα production by alveolar macrophages at concentrations as low as 0.1–10 nM (figure 1B), a concentration range that appears to be clinically relevant. In guinea pigs, inhaled salmeterol (0.12–12 mM) and salbutamol (0.2 and 2 mM) strongly inhibited histamine-induced bronchoconstriction which was argued to be of predictive value in terms of relative potencies and durations of action of inhaled β2 agonists in man [33]. In the present study the doses of salbutamol and salmeterol (both 2.4 mM) were well within the effective range as tested in guinea pigs, and proved to be effective and propranolol sensitive (β receptor dependent) with respect to inhibition of both TNFα production and bacterial clearance after NTHi challenge. Together, these data suggest that salmeterol and salbutamol as nebulized in our model is present in lungs in sufficiently high topical and probably clinically relevant concentrations to cause β-adrenoceptor dependent inhibition of TNFα production and bacterial clearance in NTHi infected lungs.

It should be noted that our studies with salbutamol were focused on the most relevant time point of the time course studies using salmeterol. In addition, we did not determine the effect of propranolol in salbutamol treated mice. The salbutamol studies were done to exclude a salmeterol specific effect and to show that the effects observed were specific for the class of β2 agonists. Considering that both salmeterol and salbutamol inhibited the clearance of NTHi, our investigation provides proof for an effect that indeed is mediated by stimulation of β2 receptors.

Salmeterol and salbutamol consistently delayed the clearance of NTHi from the lungs, a finding that was reproduced in several experiments (figure 5A, 5B and 6). Considering that the β2 agonist induced inhibition of early TNFα release can not explain the adverse effect of salmeterol and salbutamol on the bacterial clearance, and considering that these agents did not influence neutrophil recruitment, other mechanisms must be involved. In this respect it should be noted that β2 agonists can inhibit several inflammatory cell functions considered important for defense against bacteria. For instance neutrophil respiratory burst activity [34] and exocytosis [35] were shown to be attenuated by β2 agonist treatment. Additionally, bacterial killing and superoxide anion release by alveolar macrophages was strongly suppressed by both salbutamol and formoterol [36]. In contrast, no effect of β2 agonists on phagocytosis by neutrophils and alveolar macrophages was observed [37,38]. Other studies have documented possible protective effects of β2 agonists on respiratory epithelium. In particular, preincubation of human nasal turbinates with salmeterol attenuated *H. influenzae *reduced epithelial damage without influencing the total number of bacteria adhering to the organ culture [39]. Similar observations have been made in nasal turbinate cultures infected with *P. aeruginosa *[40].

## Conclusion

Our study suggests that, at least in mice, inhalation of β2 agonists impairs the clearance of NTHi from the airways. Obviously, these data do not imply that the use of β2 agonists should be discouraged in patients with obstructive pulmonary diseases; rather they exemplify the complex anti-inflammatory actions of β2 agonists in the pulmonary compartment and that a potential role in the suppression of pulmonary antibacterial defenses must not be overlooked.

## Competing interests

The author(s) declare that they have no competing interests.

## Authors' contributions

NA participated in the design of the studies, performed the studies, analyzed the data and wrote the first draft of manuscript. SF was responsible for the performance and analysis of the histopathology and took part in writing the manuscript. CV and AFV supervised the laboratory analyses and took part in writing the manuscript. WB contributed vital reagents and took part in writing the manuscript. HMJ took part in designing the studies and writing the manuscript. TvdP designed and supervised the project and wrote the final version of the manuscript.
